# Dual respiratory virus detection in adult patients with acute respiratory illness

**DOI:** 10.1186/s12879-021-06699-z

**Published:** 2021-09-23

**Authors:** Yu-Mi Lee, Tark Kim, Ki-Ho Park, Seong-Ho Choi, Yee Gyung Kwak, Eun Ju Choo, Jin-Won Chung, Mi Suk Lee

**Affiliations:** 1 Division of Infectious Diseases, Department of Internal Medicine, Kyung Hee University Hospital, Kyung Hee University School of Medicine, Seoul, Republic of Korea; 2 Division of Infectious Diseases, Department of Internal Medicine, Soonchunhyang University Bucheon Hospital, Bucheon, Republic of Korea; 3 Division of Infectious Diseases, Department of Internal Medicine, Chung-Ang University Hospital, Chung-Ang University College of Medicine, 102 Heukseok-ro, Dongjak-gu, Seoul, 06973 Republic of Korea; 4 Division of Infectious Diseases, Department of Internal Medicine, Inje University Ilsan Paik Hospital, Goyang, Republic of Korea

**Keywords:** Adult, Respiratory tract infections, Viruses

## Abstract

**Background:**

Nonrandom multiple respiratory virus (RV) detection provides evidence for viral interference among respiratory viruses. However, little is known as to whether it occurs randomly.

**Methods:**

The prevalence of dual RV detection (DRVD) in patients with acute respiratory illnesses (ARIs) at 4 academic medical centers was investigated; data about the prevalence of 8 RVs were collected from the Korean national RV surveillance dataset. Linear regression analysis was performed to assess the correlation between observed and estimated prevalence of each type of DRVD.

**Results:**

In total, 108 patients with ARIs showing DRVD were included in this study between 2011 and 2017. In several types of regression analysis, a strong correlation was observed between the observed and estimated prevalence of each type of DRVD. Excluding three DRVD types (influenza/picornavirus, influenza/human metapneumovirus, and adenovirus/respiratory syncytial virus), the slope of the regression line was higher than that of the line of random occurrence (1.231 > 1.000) and the 95% confidence interval of the regression line was located above the line of random occurrence.

**Conclusions:**

Contrary to the results of previous epidemiologic studies, most types of DRVD occur more frequently than expected from the prevalence rates of individual RV, except for three underrepresented pairs above.

## Background

After performing multiplex polymerase chain reaction (PCR) test for the detection of common respiratory viruses (RVs), simultaneous detection of two different RVs—dual RV detection (DRVD)—is frequently observed in patients with acute respiratory illness (ARI). Clinicians are interested in the two aspects of DRVD. First, patients with ARI showing DRVD may present more severe clinical manifestations or poorer clinical outcomes than those showing single RV detection. Many clinical studies have been conducted for this subject [1, 2]. Second, an observed prevalence of a certain type of DRVD may provide an indication to the interaction between the two different RVs. For example, if the observed prevalence would be lower or higher than an expected prevalence estimated from the prevalence rates of individual RVs (i.e., nonrandom occurrence of DRVD), the two RVs are likely to have an interfering or enhancing relationship with each other. A small number of epidemiological studies explored this topic and reported that many types of DRVD were underrepresented when the prevalence rates of the involved RVs were considered [3–5]. These studies support the presence of viral interference between RVs. However, they have an important problem in their methodology. Estimation of the prevalence of DRVD based on the average prevalence rates of the individual RVs may be biased because outbreaks of two different RVs can occur either with overlap or without overlap. For example, DRVD may be frequently observed when the outbreaks of two RVs occur with overlap; in contrast, it may be infrequently encountered when the outbreaks occur without overlap. Thus, to determine the ever-changing overlap between outbreaks of two RVs, estimation on the prevalence of DRVD should be performed per each short time interval rather than performed once across the entire study period. In addition to the problem, most of these epidemiological studies were conducted in children and, even when targeting all age groups, it was rare to present data on adult patients separately. Furthermore, since their study periods ranged only within a year, their results have poor generalizability.

Therefore, we estimated the frequency of DRVD on a weekly basis from the national RV surveillance dataset that included the weekly prevalence of each RV in the community over a much longer study period of about 6 years (the surveillance dataset) and summed the frequencies according to each type of DRVD. Moreover, we collected the clinical data of adult hospitalized patients with ARI showing every type of DRVD from multiple academic medical centers (the clinical dataset). Subsequently, we compared the observed frequency of each type of DRVD from the clinical dataset to the summed weekly estimated frequency of each corresponding DRVD type from the surveillance dataset to identify if DRVD occurs randomly or not.

## Methods

### Collection of the clinical dataset

This study was performed at 4 academic medical centers in South Korea: 2 in Seoul (Chung-Ang University Hospital and Kyung Hee University Medical Center) and 2 in the Province of Gyeonggi (Inje University Ilsan Paik Hospital and Soonchunhyang University Bucheon Hospital). We identified adult patients (aged ≥ 16 years) with ARI who underwent multiplex PCR test for RVs and showed DRVD during the following periods at each study center: Chung-Ang University Hospital, first week of 2012–14th week of 2016; Kyung Hee University Medical Center, first week of 2011–13th week of 2017; Inje University Ilsan Paik Hospital, first week of 2012–14th week of 2017; and Soonchunhyang University Bucheon Hospital, first week of 2012–43rd week of 2015. Infectious disease physicians at each center retrospectively reviewed the patients’ medical records and chest radiographs. Demographic characteristics, date of multiplex PCR test, illness-related symptoms, and chest radiography findings were investigated.

#### ***Definition***

RVs were classified into the following 8 categories: adenovirus (ADV), human bocavirus (hBoV), human coronavirus (hCoV; hCoV-229E, hCoV-NL63, hCoV-OC43, and hCoV-HKU1 were included), influenza virus (IFV; IFV-A and IFV-B were included), human metapneumovirus (hMPV), parainfluenza virus (PIV; PIV-1, PIV-2, PIV-3, and PIV-4 were included), picornavirus (Picor; rhinovirus [RHV] and enterovirus [EV] were included), and respiratory syncytial virus (RSV; RSV-A and RSV-B were included). ARI was defined as the development of at least one of the following respiratory symptoms: cough, sputum production, rhinorrhea, sore throat, and dyspnea.

### Collection of the surveillance dataset

The national RV surveillance dataset of South Korea, that is, the data from the Korea Influenza and Respiratory Surveillance System established by the Korean Center for Disease Control and Prevention (KCDC), was used to determine the viral activity of each of the 8 RVs in the community. The dataset included weekly reports of positivity rates for each of the 8 RVs: ADV, hBoV, hCoV, IFV, hMPV, PIV, RHV, and RSV. Detailed information about the dataset can be found elsewhere [6]. Data pertaining to the study period (from the first week of 2011 to the 14th week of 2017 [a total of 327 weeks]) were retrieved from the KCDC website [7].

### Estimation of frequency of dual respiratory virus detection from the surveillance dataset

**Fig. 1:**
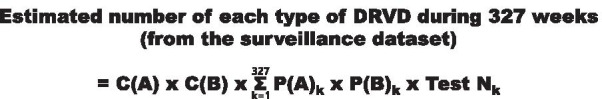
Formula for the estimation of the number of adult hospitalized patients with acute respiratory illnesses who were simultaneously detected as having both respiratory viruses A and B according to the Korean national respiratory virus surveillance dataset. DRVD, dual respiratory virus detection; C(A), conversion constant of respiratory virus A; C(B), conversion constant of respiratory virus B; P(A)_k_, prevalence of respiratory virus A during k week according to the surveillance dataset; P(B)_k_, prevalence of respiratory virus B during k week according to the surveillance dataset; Test N_k_, number of polymerase chain reaction tests performed during k week at the study centers

**Fig. 2:**
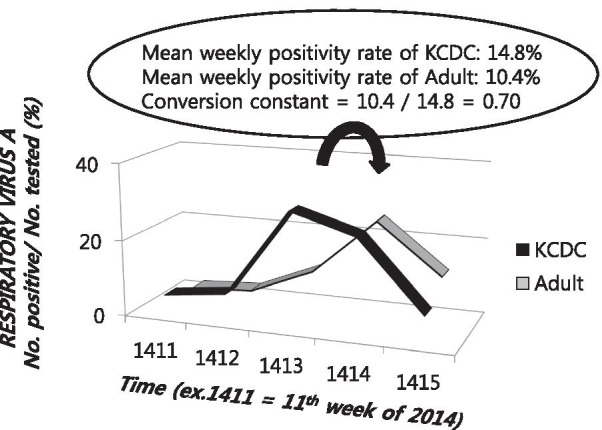
An illustrated example that shows the formation process of conversion constant regarding “respiratory virus A” between the Koran Center for Disease Control dataset and a single university hospital dataset of adult patients with acute respiratory illness (adult). For each RV, conversion constant was calculated from the data of the previous study of our group

Estimation of the number of patients who showed DRVD of RVs A and B during the 327 weeks was performed as described in Fig. 1. The prevalence of each RV during k week (P[A]_k_ and P[B]_k_) was obtained from the surveillance dataset, number of multiplex PCR tests performed for RVs during k week (N_k_) was available from the study centers, and conversion constants for each RV (C[A] and C[B]) were obtained from a previous study conducted by our group, which showed the relationship between the surveillance dataset and the data of adult hospitalized patients with ARI during 2 years [6]. The conversion constant was a ratio of the mean weekly positivity rate of a specific RV according to hospital data to the mean weekly positivity rate of the same RV according to the surveillance data (Fig. 2). The followings are the conversion constants for the 8 RV categories: ADV, 0.373; hBoV, 0.354; hCoV, 0.480; IFV, 0.410; hMPV, 0.570; PIV, 0.181; Picor, 0.386; and RSV, 0.690. For each of the 327 weeks, frequencies of the 28 types of DRVD were calculated. The frequencies were summed up for each 28 types of DRVD.

### Statistical analyses

Linear regression analysis was performed to assess the correlation between the observed and estimated prevalence rates of each type of DRVD. We used the standard model of linear regression, assuming the presence of a dependent variable, *Y* (observed frequency according to the clinical dataset), and an independent variable, *X* (estimated frequency according to the surveillance dataset). *P* value < 0.05 was considered statistically significant. All statistical analyses were performed using Statistical Package for the Social Sciences (SPSS) software (version 18.0; SPSS Inc., Chicago, IL, USA).

## Results

During the study period, 5,476 RV-related multiplex PCR tests were performed at the 4 study centers, and the median number of tests performed each week was 14.0 (interquartile range, 7.0–24.0). A total of 134 adult hospitalized patients showed multiple RV detection. Of these patients, 10 had no symptoms and signs of ARI. Of the remaining 124 patients, 10 showed DRVD involving the same RV groups (IFV-A/IFV-B, 3; hCoV-NL63/hCoV-OC43, 2; hCoV-229E/hCoV-OC43, 2; PIV-1/PIV-3, 2; and RHV/EV, 1), and 6 showed detection of 3 RVs (ADV/RHV/EV, IFV/RHV/RSV, hCoV/IFV/PIV, ADV/hCoV/RSV, ADV/IFV/RHV, and ADV/hBoV/hCoV). After excluding these patients, 108 adult hospitalized patients with ARIs showing DRVD were finally included in our analyses. The median age of the study patients was 68.5 years (ranged from 16 to 94 years) and more than a half of them were male (55, 50.9%). The most common underlying disease was diabetes mellitus (39, 36.1%), followed by chronic lung disease (26, 24.1%), chronic renal disease (20, 18.5%), use of immunosuppressants (15, 13.9%), cerebrovascular disease (14, 13.0%), heart failure (13, 12.0%), solid tumor (12, 11.1%), and so on. The majority of them had community-acquired ARI (95, 88.0%). Pneumonia, severe pneumonia (pneumonia requiring the use of ventilator therapy or vasopressor), co-infection with non-RV pathogens, and in-hospital death were observed in 87 (80.6%), 35 (32.4%), 48 (44.4%), and 18 (17.5% of 103), respectively. Table 1 shows the types of DRVD observed among the included patients. hCoV/IFV was the most common DRVD type (11.4%). Of the 28 types of DRVD, which could be inferred from the 8 RV categories, hBoV/hCoV, hBoV/hMPV, hBoV/PIV, and hMPV/RSV were not observed at all.

**Table 1:** Distribution of the types of dual respiratory virus detection (DRVD) among the 108 adult hospitalized patients with acute respiratory illnesses at 4 academic medical centers and estimated numbers of each DRVD types before and after multiplying by conversion constants

Type of DRVD	Numbers (%) of patients	Estimated number of patients with DRVD from surveillance dataset (before multiplying by conversion constants) (column A)	Estimated number of patients with DRVD from surveillance dataset (after multiplying by conversion constants) (column B)
hCoV/IFV	13 (12.0)	45.6	9.0
ADV/Picor	10 (9.3)	53.1	7.7
Picor/RSV	9 (8.3)	31.6	8.4
ADV/IFV	9 (8.3)	51.6	7.9
IFV/Picor	9 (8.3)	97.7	15.5
hCoV/RSV	7 (6.5)	13.9	4.6
hMPV/Picor	7 (6.5)	20.6	4.5
IFV/RSV	7 (6.5)	25.8	7.3
ADV/hBoV	5 (4.6)	7.7	1.0
hCoV/Picor	4 (3.7)	29.3	5.4
PIV/Picor	4 (3.7)	49.6	3.5
IFV/PIV	3 (2.8)	21.0	1.6
ADV/hCoV	3 (2.8)	12.7	2.3
hBoV/IFV	3 (2.8)	11.8	1.7
hBoV/Picor	3 (2.8)	17.5	2.4
ADV/hMPV	2 (1.9)	7.0	1.5
ADV/PIV	2 (1.9)	20.4	1.4
IFV/hMPV	2 (1.9)	28.3	6.6
ADV/RSV	1 (0.9)	13.8	3.6
hBoV/ RSV	1 (0.9)	1.4	0.3
hCoV/hMPV	1 (0.9)	6.4	1.8
hCoV/PIV	1 (0.9)	6.8	0.6
hMPV/PIV	1 (0.9)	7.8	0.8
PIV/RSV	1 (0.9)	5.5	0.7
hBoV/hCoV	0	2.5	0.4
hBoV/hMPV	0	3.9	0.8
hBoV/PIV	0	10.4	0.7
hMPV/RSV	0	3.4	1.3

*ADV* adenovirus, *hBoV* human bocavirus, *hCoV* human coronavirus, *IFV* influenza virus, *hMPV* human metapneumovirus, *PIV* parainfluenza virus, *Picor* picornavirus, *RSV* respiratory syncytial virus

**Fig. 3:**
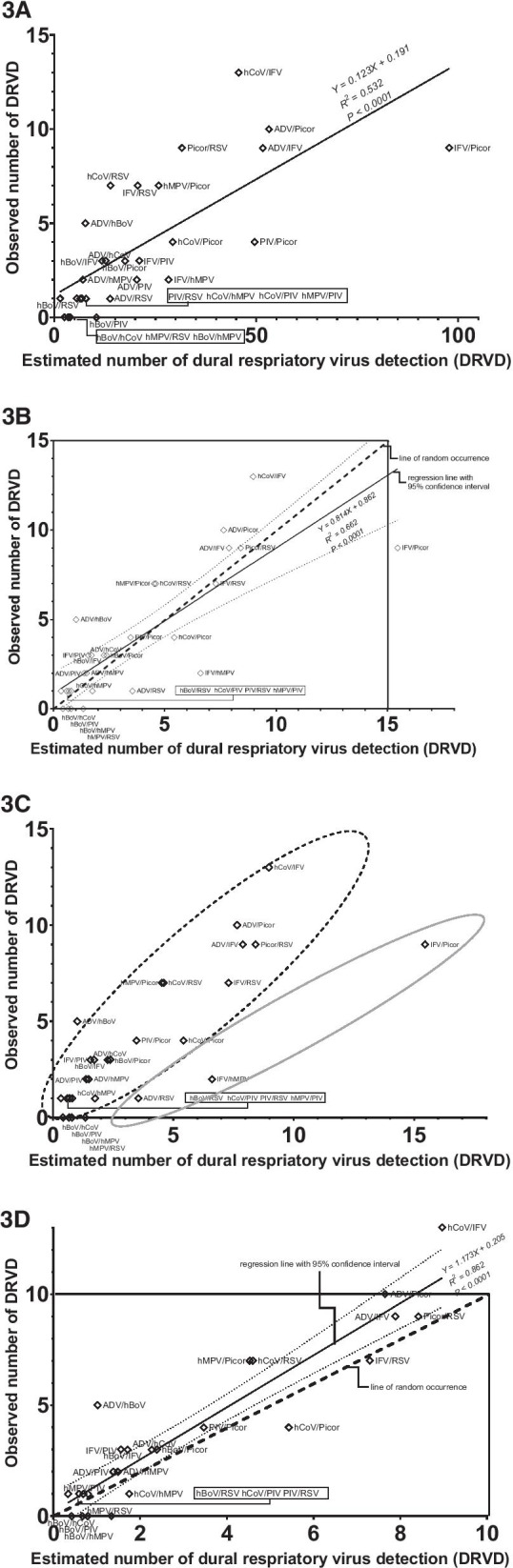
Scatter plots showing the correlation between the observed and estimated frequency of dual respiratory virus detection with the regression line and 95% confidence intervals. Each hollow rhombus represents one particular DRVD pair. **A** Before multiplying by conversion constants. **B** After multiplying by conversion constants. **C** (**B**) attached with dotted and gray circles that shows two kinds of association with different slopes. **D** After removing 3 types of DRVD (presented in Fig. 3B; IFV/Picor, IFV/hMPV, and ADV/RSV) and multiplying by conversion constants. White boxes include equations of the regression lines, R-squared values, and *P* values. Dotted lines in **B**, **C** are lines showing direct proportional relationship, that is, lines of random occurrence (*Y* = *X*)

Figure 3 shows regression analysis between the observed numbers of different DRVD types and the estimated numbers of corresponding DRVD types described in Table 1.

Figure 3A is a scatter plot showing the correlation between the observed and the estimated numbers of DRVD. Estimated numbers here are values before the step of multiplication by conversion constants (column A in Table 1). The significant positive correlation is presented between the observed and the estimated numbers (*R*^*2*^ = 0.532, *P* < 0.0001).

Figure 3B is another scatter plot showing the correlation between the observed and the estimated numbers of DRVD. Estimated numbers here are values after the step of multiplication by conversion constants (column B in Table 1). The observed numbers are also significantly positively correlated with the estimated numbers (*R*^*2*^ = 0.662, *P* < 0.0001). The line showing direct proportional relationship (thick dotted line; the line of random occurrence, *Y* = *X*) is within the 95% confidence intervals of the regression line (*Y* = 0.814*X* + 0.862). Figure 3C is the same scatter plot as Fig. 3B. In Fig. 3C, there are two types of associations that appear different in slopes (marked with black dotted circle and gray solid circle respectively). Three types of DRVD—IFV/Picor, IFV/hMPV, and ADV/RSV—were included in the gray solid circle with lower slope.

Figure 3D is the other scatter plot excluding the 3 types of DRVD in the gray solid circle from Fig. 3C. A significant positive correlation was also observed between the observed numbers and the estimated numbers (*R*^*2*^ = 0.862, *P* < 0.0001). After the removal of the above 3 DRVD types from Fig. 3C, the slope of the regression line (*Y* = 1.173*X* + 0.205) was higher than that (*Y* = 0.814*X* + 0.862) in Fig. 3B, and most parts of the line of random occurrence (thick dotted line, *Y* = *X*) lies below and outside the 95% confidence intervals of the regression line.

## Discussion

The observed frequencies of the 28 types of DRVD from several hospitals and the estimated frequencies of each corresponding DRVD type from the national weekly RV surveillance dataset were strongly positively correlated. Most of the DRVD types occurred more frequently than expected from the prevalence rates of individual RVs except for the 3 DRVD types (IFV/Picor, IFV/hMPV, and ADV/RSV), which occurred far less frequently than expected.

Viral interaction between RVs has been suggested largely through four types of studies [8]. First, the epidemiologic analysis of outbreak curves of different RVs suggested the presence of viral interaction for a long time [9, 10]. Since the 2009 H1N1 pandemic, similar reports from surveillance datasets have been reported [11–14]. Most of the clinical evidences regarding viral interaction originated from this type of study. Second, reduction of an RV infection through vaccination was accompanied by an increased risk of other RV infections. For example, the subsequent occurrence of non-influenza RV infection increased more in patients who had been vaccinated against influenza compared to those who had not been vaccinated [15]. Third, there were a limited number of in vitro studies using RV coinfection models [16–18]. Fourth, the observed prevalence of multiple RV detection was investigated as described above since the advent of multiplex RV testing [3–5]. It is difficult to directly compare the results of the above four types due to a variety of unique variables to be considered for each type of study. The representative reports of the fourth type of study did not consider the impact of overlap between the outbreaks of RVs as described above, but it was considered in this study. Considering the heterogeneity across the previous studies, the results of this study were different from the previous studies; thus, they are notable.

Most DRVD types were found more frequently than expected from the prevalence rates of individual RVs. These findings are contradictory to the findings of the previous epidemiological studies, which reported that many types of DRVD were underrepresented. Brunstein et al. reported that IFV-B/RSV-A, hMPV/RHV, RSV-A/hMPV, and RSV-B/hMPV were the underrepresented pairs [3]. Greer et al. reported that ADV/Picor, hBoV/hMPV, hBoV/Picor, hBoV/RSV, hCoV/Picor, IFV/Picor, hMPV/Picor, PIV/Picor, and RSV/Picor were the underrepresented pairs [4]. According to the study by Tanner et al., some pairs were suggested to be underrepresented (IFV/hMPV, IFV/RHV, PIV/RHV, and RSV/RHV), whereas some pairs were suggested to be overrepresented (ADV/PIV, ADV/RHV, and ADV/RSV) [5]. However, any type of DRVD was not found to be consistently underrepresented across these 3 studies. This inconsistency may be a result from the aforementioned limitations of these previous studies. Our national surveillance dataset included information about > 6 years of RV activity and of > 10,000 RV-related PCR tests that were performed by the KCDC every year [6]. The clinical dataset was also collected for a 4–6-year period from multiple academic medical centers. Thus, we propose that RVs are co-detected more frequently than expected in adult hospitalized patients with ARI, except for several pairs of RVs. We do not know which factors of host immunity, viral pathogenesis, and their interactions influenced the results. However, increased host susceptibility and prolonged viral shedding, which may be found more frequently in hospitalized patients than in patients in the community, at least in part, may affect the overrepresentation of DRVD in this study.

Three DRVD types (IFV/Picor, IFV/hMPV, and ADV/RSV) were removed from Fig. 3B because they showed a trend of association different from that of the other 25 types, as shown in Fig. 3C. Viral interference between IFV and Picor has been suggested from many types of studies. The outbreak of IFV rarely overlapped with that of RHV from the analyses of outbreak curves [11, 12]. IFV-vaccinated patients had been infected with RHV more frequently than those who had not been IFV-vaccinated [15]. IFV/Picor was reported to be underrepresented considering the prevalence rates of the individual RVs in the studies of Greer et al. and Tanner et al. [4, 5]. Considering these and our results, it is suggested that IFV and Picor neither co-circulate easily nor are co-detected easily even when they co-circulate. Viral interference between the other two DRVD types (IFV/hMPV and ADV/RSV) has not been reported. It may be due to their relatively infrequent occurrence compared to other types of DRVD, as shown in Table 1. These findings should be validated by other external sources.

This study has important limitations. Surveillance dataset and clinical dataset were collected with different purposes, objects, and methods. Because of the differences, care should be taken when interpreting the results. However, methodological limitations of the previous studies may not be easily overcome without such vast resources of surveillance dataset. Additionally, we used the data of the previous study of our group to address this problem—the conversion constants were calculated from the data of the study [6]. This study included a relatively large number of patients for 108 weeks and was conducted in one of the areas (Seoul) as the study site and under the same research condition. The other limitation is that it was difficult to confirm whether the multiplex RT-PCR method used in the study hospitals and the laboratories affiliated with the KCDC was the same method.

## Conclusion

We found that, in the era of multiplex PCR test for RVs, most DRVD types significantly occurred more frequently than expected from the prevalence rates of individual RVs, except for a number of underrepresented pairs such as IFV/Picor, IFV/hMPV, and ADV/RSV.

## Data Availability

The datasets used and/or analyzed during the current study are available from the corresponding author on reasonable request.

## Abbreviations

ADV: Adenovirus
ARI: Acute respiratory illness
DRVD: Dual respiratory virus detection
EV: Enterovirus
hBoV: Human bocavirus
hCoV: Human coronavirus
hMPV: Human metapneumovirus
IFV: Influenza virus
KCDC: Korean Center for Disease Control and Prevention
Picor: Picornavirus
PIV: Parainfluenza virus
PCR: Polymerase chain reaction
RHV: Rhinovirus
RSV: Respiratory syncytial virus
RV: Respiratory virus
